# An Illustration is Worth Ten Thousand Words: An Extraordinary Approach to Presenting Information Through Infographics

**DOI:** 10.1007/s40670-025-02285-z

**Published:** 2025-01-28

**Authors:** Mathys J. Labuschagne, Isabella du Preez, Helena Prior Filipe

**Affiliations:** 1 Clinical Simulation and Skills Unit, School of Biomedical Sciences, Faculty of Health Sciences, University of the Free State, 205 Nelson Mandela Drive, Bloemfontein, 9300 South Africa; 2 School of Clinical Medicine, Research and Development, Faculty of Health Sciences, University of the Free State, Bloemfontein, South Africa; 3 West Lisbon Hospitals Center, Hospital Egas Moniz, Lisbon, Portugal; 4 Egas Moniz Center for Interdisciplinary Research, Lisbon, Portugal

**Keywords:** Infographics, Simulation-based education, Ophthalmology, Just-in-Time Teaching (JiTT), Visual design principles, Clinical teaching tools

## Abstract

**Introduction:**

Infographics summarise concepts visually for quick reference. Four infographics, inspired by articles on simulation-based education for ophthalmologists, were created to simplify complex ideas. The study evaluated the infographics’ effectiveness aligning with published recommendations to make simulation education user-friendly and practical, supporting busy ophthalmologist educators in designing and facilitating simulation training sessions.

**Methods:**

This descriptive cross-sectional study evaluated the infographics created by the second author, focussing on their quality, visual appeal, and effectiveness in conveying the key messages to be used for microlearning and Just-in-Time Teaching (JiTT). An evidence-based electronic questionnaire was used to evaluate design principles of effective infographics. The same questions concerning each infographic were presented to ophthalmologist educators and graphic designers.

**Results:**

Feedback informed recommendations for infographics to define a target audience, highlight the heading, and ensure a clear narrative with an identifiable key message. Content should simplify complex concepts and be applicable to teaching. Include accessible references, limited colours and fonts, and logical alignment prioritising key elements. Use simple imagery and effective charts. Add a digital object identifier (DOI) for citation and discovery. Infographics can be shared via journals and social media, or used as Just-in-Time Teaching (JiTT) tools to support professional development and simulation teaching preparation.

**Conclusion:**

Effectively designed infographics as pragmatic focused graphic storytelling tools can support clinical educators in their daily simulation-based teaching activities and JiTT. This article provides tips on designing infographics for this purpose.

## Introduction

The dissemination of information to a broader audience can be challenging, particularly when time is limited and the volume of information is substantial. However, in the contemporary era, innovative approaches to sharing information in health education have emerged [1]. One such approach to optimise the communication of information in a visual method is infographics, which have become increasingly popular for enhancing the impact of the information [2]. An infographic, a term combining “information” and “graphic” [3], is used for data visualisation, presenting information through graphs and diagrams [4]. Besides these visual components, infographics also include related text to provide context and additional information on the subject [5].

Infographics serve as graphic depictions of material, facilitating the communication of intricate information, distribution of scientific findings, and promotion of alternative approaches to day-to-day practices [6]. With the aim of providing an overview of simulation-based education best practices, the authors and their fellow colleagues of the Ophthalmology Foundation Simulation-Based Education Subcommittee (https://ophthalmologyfoundation.org/faculty-education/simulation-based-education/) have recently published a series of four articles [7–10]. Although being inspired by simulation-based ophthalmology training, the series can be extrapolated into various healthcare specialties. In a continuum, the authors designed four infographics, each highlighting the main insight of each of the articles. This was to capture the attention of the clinical educator interested in teaching by simulation, improving understanding, and promoting simulation-based educational practices.

Consistent with the perspectives offered by Khoury et al. [11], this study involved a collaborative partnership between simulation educators, clinicians (ophthalmologists), and a graphic designer, which culminated in the design of four infographics. Infographics can serve as a quick and easy reference guide to assist in training activities by summarising complex concepts and processes in a visual and easy-to-use format. Infographics can serve as an effective educational tool to enhance teaching strategies in fast-paced clinical environments. They can be customised for various programs and incorporated into the teaching resources used by residents and faculty for nano- and microlearning and Just-in-Time Teaching (JiTT) [12], using applications, email, social media, or paper-based.

The busy clinician educator can find it challenging to allocate time to engage in the complex interplay between competing responsibilities that involve clinical care, research, administration, and education [13, 14]. These clinical educators often rely on conventional faculty development activities to enhance their proficiency in teaching. Microlearning has been considered a key topic in workplace-based learning. Policymakers, educators, researchers, and faculty developers should explore possibilities to promote, design, and use microlearning to facilitate learning in the right direction while conveying valid knowledge with ethical consideration [15]. Faculty developers can integrate concepts from technology-enhanced learning such as online microlearning, and construct just-in-time (JiT) focused, brief, enjoyable, and effective learning experiences for ophthalmologist educators [14].

The aim of the Ophthalmology Foundation Simulation Committee is to promote global outreach, contextualisation, and the potential adaptation of the proven good practices of simulation-based education (SBE) to implement in diverse learning environments. A group of international educators who are experienced in SBE compiled a white paper on simulation education focusing on the ophthalmologist educator, with possible extrapolation for educators in other medical disciplines. A four-fold series of articles were published from that work [7–10]. The main message of each article was depicted in separate infographics to provide its overview, highlighting its main message and promulgate and improve simulation-based educational practices.

The objective of the study was to evaluate these four infographics according to the recommendations postulated in the literature [6, 16, 17] to simplify the complexities of simulation-based education, making them user-friendly and practical for ophthalmologist educators to use as microlearning tools for simulation design and facilitation. This article offers guidance on creating infographics for this purpose.

## Methods

The authors designed four infographics, each highlighting the main insight of each of the articles, to capture the attention of the clinical educator interested in teaching by simulation, improving understanding and simulation-based educational practices. Each infographic cites the corresponding article and displays a QR code that can be used to link directly to the original article.

### Study Design

**Fig. 1:**
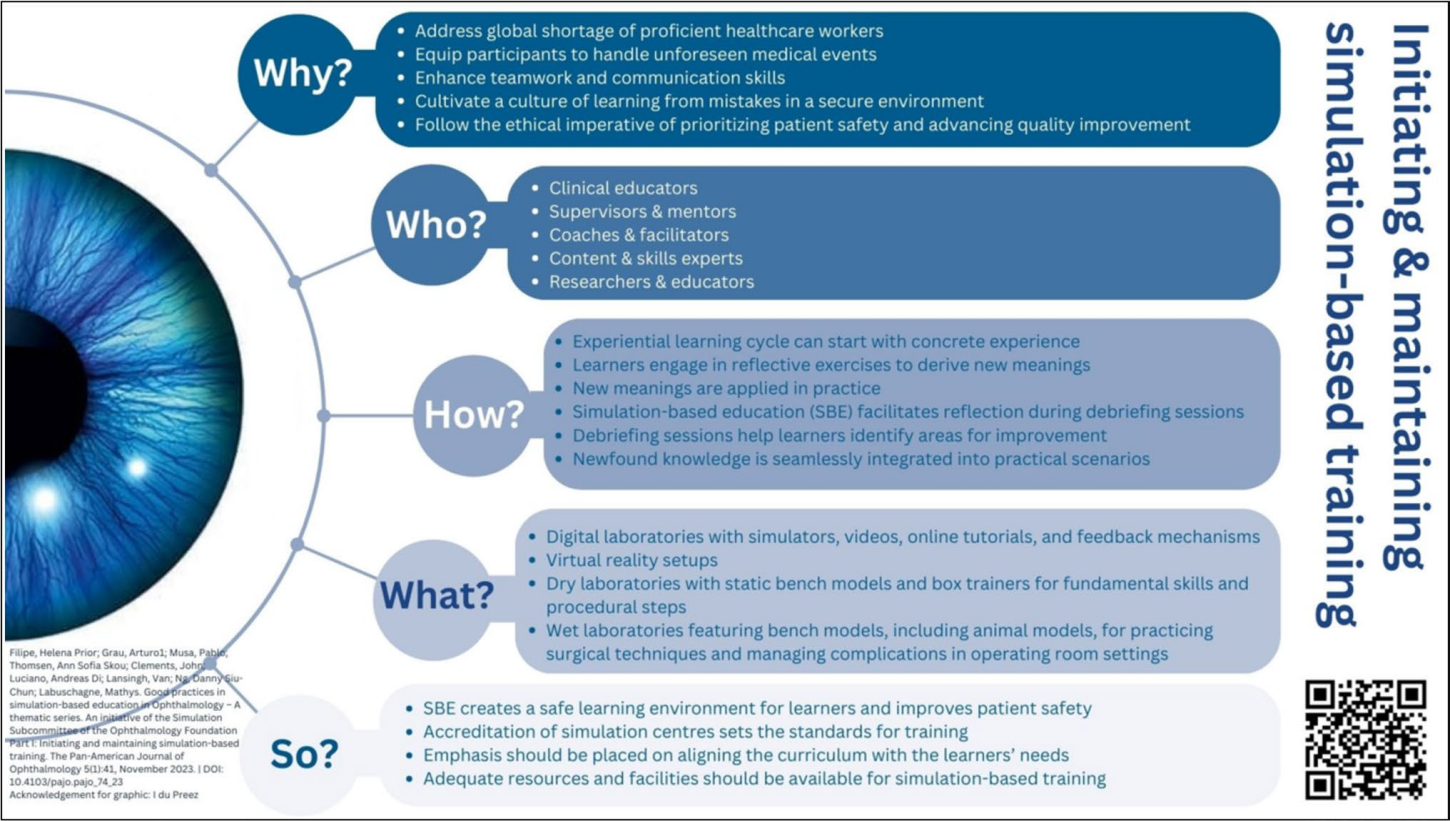
Infographic 1 [23]

**Fig. 2:**
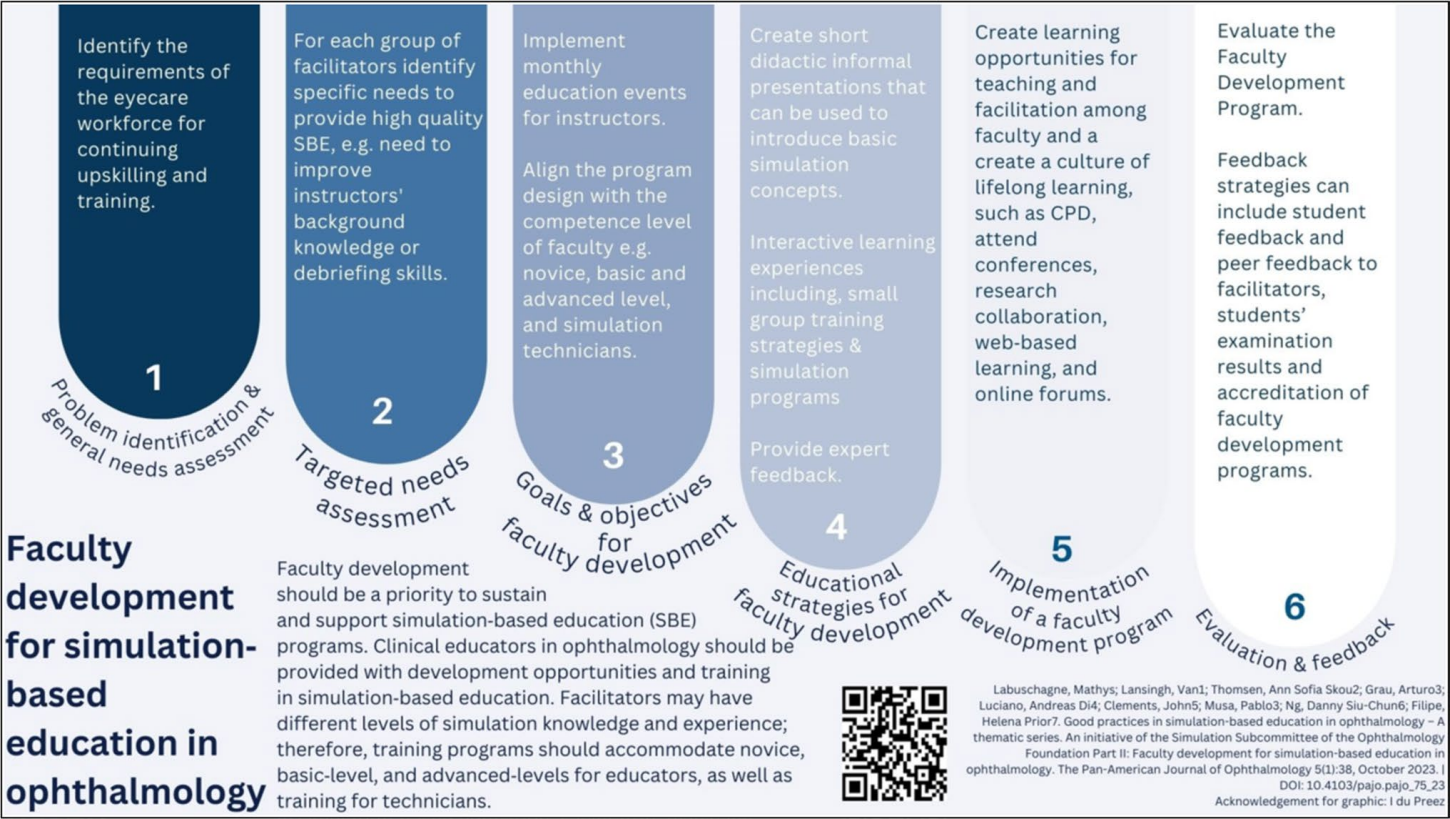
Infographic 2 [24]

**Fig. 3:**
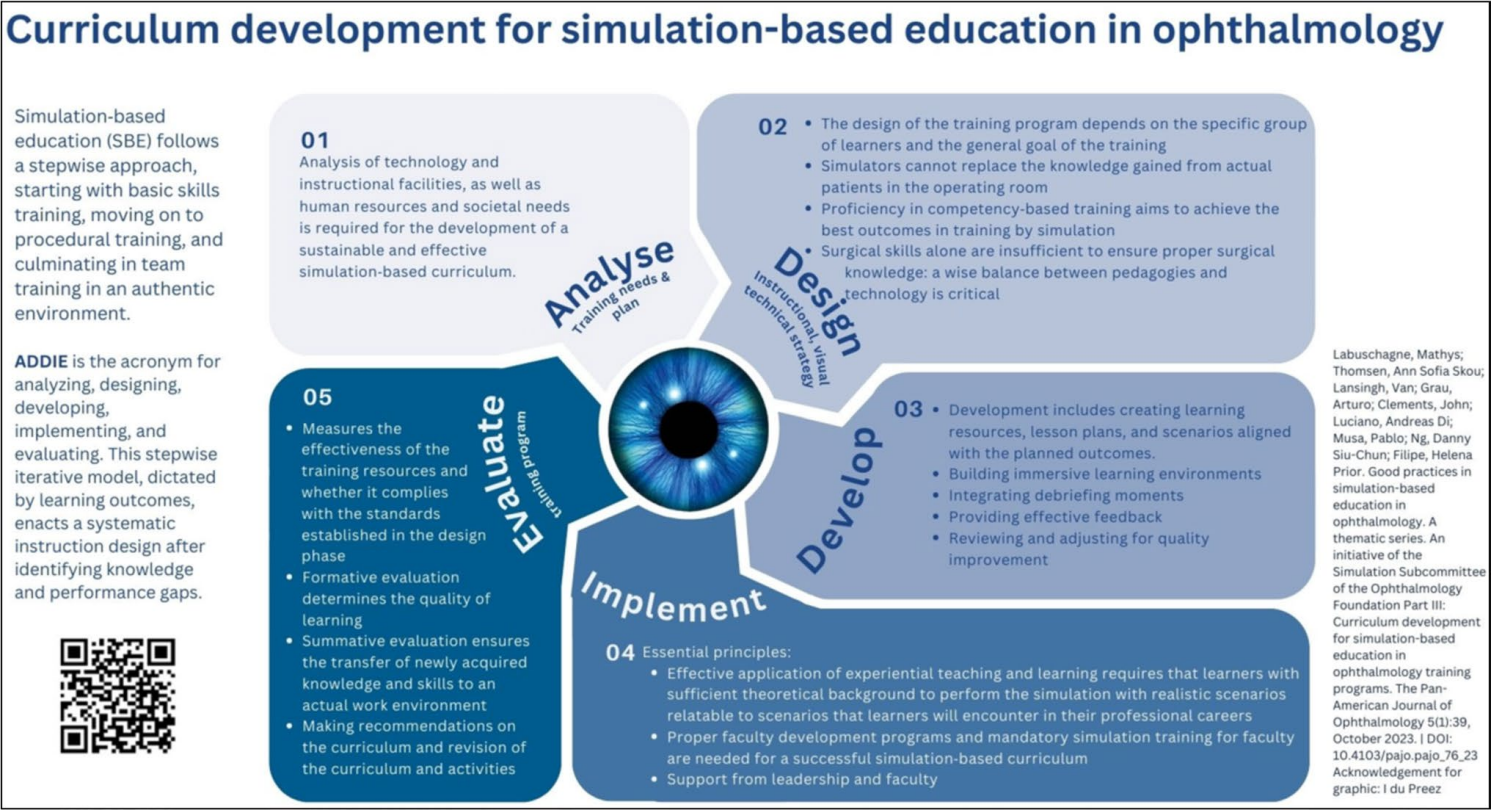
Infographic 3 [25]

**Fig. 4:**
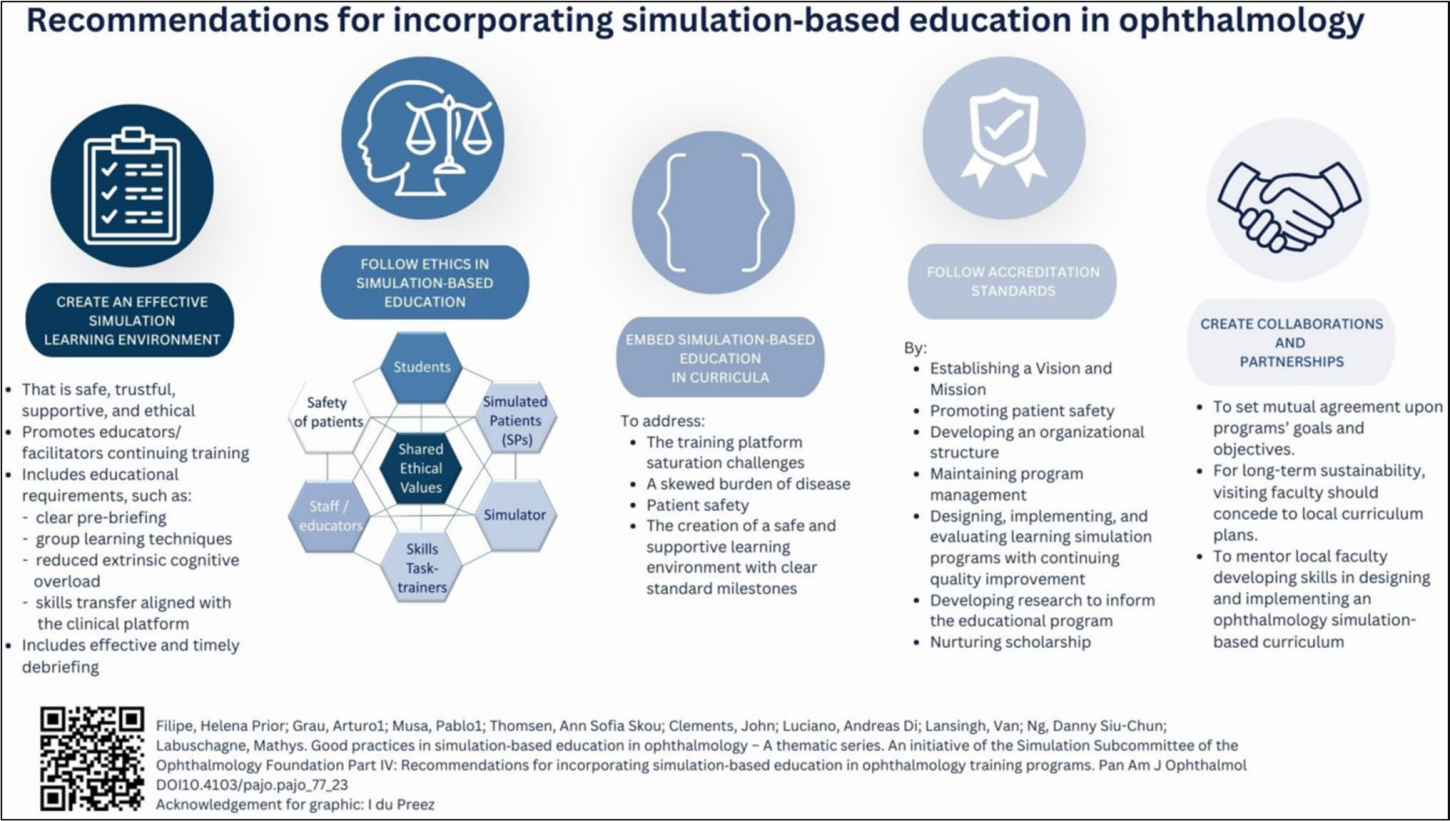
Infographic 4 [26]

A descriptive cross-sectional study design was followed to assess our four infographics regarding their (i) quality and SBE accuracy; (ii) visual appeal; and (iii) effectiveness in conveying the main takeaway message(s). An electronic questionnaire was designed based on literature regarding the general design principles of effective infographics [6, 11, 16, 17]. The same questions were consecutively asked concerning each infographic (Figs. 1, 2, 3, and 4).

### Infographic Design

The objective for each of the infographics was established and the initial design for each was prepared by the first author using PowerPoint, version 365 (Microsoft Corporation; Redmond, WA, USA). Feedback from all the authors of the articles was obtained, and comments were incorporated to refine the work. As part of the groundwork, the designs were submitted for language editing. A graphic designer (IdP) further refined the PowerPoint drafts and converted them into infographics using the Canva Professional design tool [18] to compile the infographics.

In the development and design of the four infographics, the authors employed several principles including the simple three-part story format comprising an introduction, key message, and conclusion [19]. Compelling titles were used to attract the reader, and the narrative used graphic design through organised nodes of information, arrows, and symbols to create a clear story. To provide an overview of each published article, we prioritised and emphasised the key messages playing with geometric forms, letter fonts and sizes, and hues [20].

Images and text were balanced using bullets and brief annotations and limiting text to the most important information. We limited the number of colours to three complementary colours and used contrasting colours between text and images. To ensure high readability, we used Canva Sans font type [6, 16, 17, 20].

To add validity and credibility for both lay and medical audiences, the infographics are informed by four evidence-based publications [7–10]. Mayer’s multimedia learning principles stipulate that images and words are more effective than words alone, and that learning is optimised when words and pictures are used in combination, rather than words alone [21, 22]. The identified target population was ophthalmologist educators, and all academics interested in developing good practices in simulation-based education.

The principles of infographic design were employed and each of the infographics was saved on the figshare repository of the University of the Free State in Bloemfontein, South Africa. A digital object identifier (DOI) was created for each infographic before sharing it on the various platforms. The dissemination plan for our infographics includes the organisational and monthly newsletter, as well as the University’s official social media platform and the authors’ professional social networks. Additionally, these infographics can be used in microlearning strategies in JiTT applications for faculty and trainees to be used in daily simulation teaching.

### Sampling

Purposive sampling consisted of twenty members of the Simulation Subcommittee and the Education Committee of the Ophthalmology Foundation. All are international ophthalmologists involved with education and simulation training. The questionnaire was also distributed to nine experienced graphic designers for input regarding the graphic design aspects.

### Questionnaire

Ophthalmologist educators and graphic designers were requested to complete the questionnaire pertaining to the infographics, to obtain information and refine our product before a wider dissemination. The electronic questionnaire was available on REDCap (REDCap Consortium; Fort Lauderdale, FL, USA) via a link provided to the participants. The questionnaire displayed the infographics with questions related to the general principles in infographic design, such as target audience, heading of the infographic, narrative, key message and objective of the infographic [16], and transferability of the infographic’s information to educational practice. The participants had to rate the graphic design principles applied to the infographics on a 5-point Likert scale, where 1 represented “poor”, 3 “neutral”, and 5 “good”. These design principles included the use of colours and fonts, alignment of the elements, prioritisation of certain parts by adding elements representing emphasis, the use of imagery in the infographic, and the selection of charts and graphs [6, 11, 17].

### Statistical Analysis

Basic descriptive statistical analysis was performed. Data were analysed by the authors using frequencies and percentages for categorical variables and means and standard deviations for numerical data.

### Ethical Considerations

Ethical approval was obtained from the Health Sciences Research Ethics Committee (HSREC) before commencement of the study (ethics approval number UFS-HSD2024/0708/1806). Voluntary completion of the online questionnaire implied informed consent to participate. No identifiable data were collected from the participants. Thus, once the participant submitted the questionnaire, the researchers would not be able to link a response to a participant, and participants could not amend their responses or withdraw from the study once the survey had been submitted. All information was treated confidentially, and participation was voluntary and anonymous. No payment or any form of compensation was offered. Information was collected directly from the participants in English. The findings of the study were anonymously processed, and all data were deidentified.

## Results

The four infographics (Figs. 1, 2, 3, and 4) were evaluated by the participants and the results are summarised in Table 1. In total, 29 participants completed the questionnaire. No distinction was made between the ophthalmologist educators’ and the graphic designers’ responses when the data were analysed. The participants’ feedback is summarised in Table 1.

**Table 1:** Participants’ responses to questionnaire items (*n* = 29)

Questionnaire item	Infographic 1			Infographic 2			Infographic 3			Infographic 4		
	Initiating and maintaining SB training			Faculty development for SBE in ophthalmology			Curriculum development for SBE in ophthalmology			Recommendations for incorporating SBE in ophthalmology training programs		
	Yes	No	Unsure	Yes	No	Unsure	Yes	No	Unsure	Yes	No	Unsure
	*n* (%)	*n* (%)	*n* (%)	*n* (%)	*n* (%)	*n* (%)	*n* (%)	*n* (%)	*n* (%)	*n* (%)	*n* (%)	*n* (%)
Target audience is clearly identifiable	21 (72.4)	6 (20.6)	2 (6.9)	17 (58.6)	11 (37.9)	1 (3.4)	19 (65.5)	8 (27.5)	2 (6.9)	16 (55.2)	8 (27.5)	5 (17.2)
There is a clearly highlighted heading	19 (65.5)	9 (31.0)	1 (3.4)	28 (96.5)	1 (3.4)	0	23 (79.3)	5 (17.2)	1 (3.4)	25 (86.2)	3 (10.3)	1 (3.4)
Narrative through lines and arrows	24 (82.8)	2 (6.9)	2 (6.9)	20 (69.0)	8 (27.6)	1 (3.4)	16 (55.2)	7 (24.1)	6 (20.7)	19 (65.5)	8 (27.6)	2 (6.9)
Key message or objective can be identified	21 (72.4)	6 (20.6)	2 (6.9)	22 (75.9)	4 (13.8)	3 (10.3)	20 (69.0)	5 (17.2)	4 (13.8)	22 (75.9)	6 (20.7)	1 (3.4)
Transferability to day-to-day teaching practices	23 (79.3)	2 (6.9)	4 (13.8)	22 (75.9)	1 (3.4)	6 (20.7)	23 (79.3)	2 (6.9)	4 (13.8)	23 (79.3)	2 (6.9)	4 (13.8)
Linked to literature (reference)	19 (65.5)	3 (10.3)	7 (24.1)	21 (72.4)	1 (3.4)	6 (20.7)	22 (75.9)	1 (3.4)	6 (20.7)	23 (79.3)	2 (6.9)	4 (13.8)
Colours and fonts restricted to minimum	23 (79.3)	3 (10.3)	3 (10.3)	24 (82.2)	2 (6.9)	2 (6.9)	25 (86.2)	2 (6.9)	2 (6.9)	24 (82.2)	3 (10.3)	1 (3.4)

Table 1 summarises the participants’ feedback on the four infographics obtained by means of the questionnaire. The questionnaire items were related to identification of the target audience, clarity of the heading, promoting the narrative through lines and arrows, identification of the key message or objective, transferability to day-to-day teaching practices, reference to existing literature, and the use of colours and fonts.

On average, 62.9% of the participants indicated that the target audience was clearly identifiable, ranging between 55.2% (Infographic 4) and 72.4% (Infographic 1). The infographic feature that received the highest average positive response was the use of colours and fonts, with 82.5% of participants noting that colours and fonts were restricted to the minimum, followed by 81.9% who agreed that a clearly highlighted heading was present. The single feature that received the highest positive response (*n* = 28; 96.5%) was the heading of Infographic 2, while the lowest was 55.2% each for identification of the target audience on Infographic 4 and promotion of the narrative through lines and arrows on Infographic 3.

Participants were requested to rate each infographic regarding alignment of the elements, prioritised parts, imagery used, and the selection of charts and graphs. The 5-point Likert scale results are shown in Table 2. The highest median rating (5) was allocated to the use of imagery on Infographic 1. The alignment of elements received a mean rating of 4, meaning it was considered effective in all four infographics. The imagery and prioritisation of parts both received a mean value of 4. There were no graphs or charts in the four infographics, which could explain the lower mean value of 3.5 for Infographics 1 and 3, and 4 for the other two infographics.

**Table 2:** Participants’ 5-point Likert scale rating of design features of the infographics

Feature	Infographic 1			Infographic 2			Infographic 3			Infographic 4		
	Rating			Rating			Rating			Rating		
	Mean	Median	Range	Mean	Median	Range	Mean	Median	Range	Mean	Median	Range
Alignment of elements	3.85	4	2–5	3.74	4	1–5	3.86	4	2–5	3.62	4	1–5
Prioritising parts	3.52	4	2–5	3.54	4	2–5	3.64	4	2–5	3.74	4	1–5
Imagery	3.86	5	1–5	3.70	4	1–5	3.46	3.5	2–5	3.69	4	1–5
Selection of charts and graphs	3.54	3.5	1–5	3.44	4	1–5	3.57	3.5	2–5	3.54	4	1–5

1 = poor; 3 = neutral; 5 = good

**Fig. 5:**
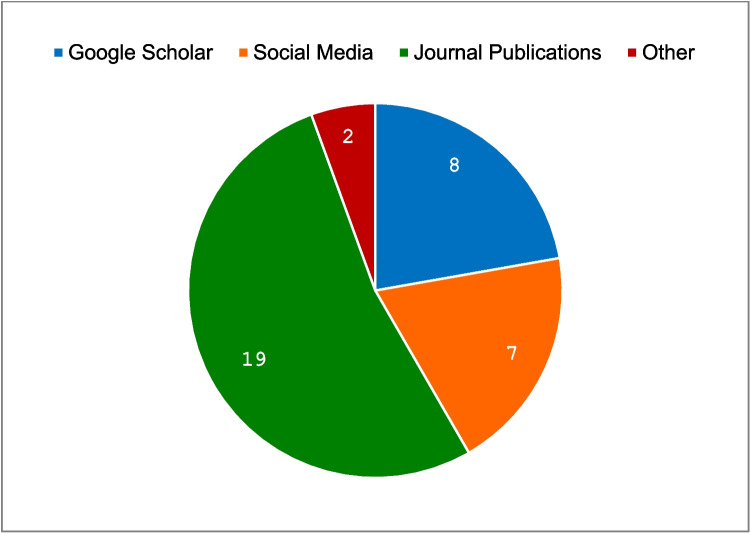
Participants’ preference of sources for finding information on infographics (*n* = 29)

Figure 5 presents the distribution of the participants’ preferences regarding sources used for obtaining information on infographics. Two-thirds (*n* = 19; 65.5%) of the participants indicated that they mostly used journal publications and/or other online sources.

## Discussion

Besides the conventional written communication through the articles [7–10], we pursued this non-traditional route of designing an infographic for each of the articles to capture the main findings, as often being done in research dissemination [15, 27]. We set out to design the infographics in a way that would attract the readers’ attention and facilitate an enjoyable, quick, and engaging read, and help to convey complex information in an understandable way. Aiming to reach the busy ophthalmologist educator tasked to juggle between clinical responsibilities and training, the authors attempted to deconstruct the complexities of simulation-based education into four infographics, each focusing on the specific topic of the published articles (initiating and maintaining SBE [7], faculty development for SBE [8], curriculum development for SBE [9], and recommendations for incorporating SBE [10]). Using visual aids such as infographics enhances comprehension when presenting information. Infographics offer a dynamic approach to conveying research findings by employing images and data visualisations such as pie charts, bar graphs, and line graphs [11].

By integrating visual material, infographics not only improve understanding but also broaden the audience reach of the research. Information conveyed through infographics tends to be better retained compared to text-only presentations [11, 21]. Moreover, articles featuring visual abstracts garner three times more views than those with text-only abstracts, consequently amplifying the alternative metrics or “altimetry” of the article [28]. Researchers are advised to actively participate in crafting both the content and design of their infographics to ensure accuracy and effectiveness [11].

Although the word “ophthalmology” appeared in three of the four titles and a clear depiction of an eye in two, between 20 and 38% of the participants felt that the target audience was not clearly identifiable. Although the target audience was ophthalmologist educators involved with SBE, the infographics were designed to be transferable to other disciplines using SBE.

The infographic with the title displayed vertically on the right-hand side of the lay-out (Infographic 1, Fig. 1) was perceived as not clearly highlighted by 31% of the participants. However, it was noteworthy that the infographic with the heading at the bottom left (Fig. 2) was still perceived as sufficient by 96.5% of the participants. The two infographics with highlighted titles at the top were perceived as sufficient by more than 80%. This was in line with the design elements identified by Naparin and Saad [20] recommending the title to be in the top centre with a bigger font size and the option of a different font type as well. The title should attract immediate attention to persuade readers to view the infographic [20]. The key messages and objectives were clearly outlined in the titles of our four infographics, as confirmed by most of the participants.

The narrative through lines and arrows was not clearly identified, and although some of the infographics made use of clear numbering and blocks of text, it seemed less effective because many participants were unsure or did not recognise the narrative. Graphic design experts recommend adding images to strengthen the narrative, while text and images must be related to the topic [20].

Transferability of the information to day-to-day teaching activities was considered good by most of the participants. Given the widespread adoption of infographics by scientific journals to amplify the visibility and adoption of their published research, infographics have emerged as a vital instrument in medical education as well [1, 29].

Ferreira et al. [17] concluded that infographics do not provide adequate information to allow readers to only rely on the information on the infographic but advise readers to read the full-text article. Therefore, we displayed the reference of the article linked to the infographic, as well as a QR code to the article, to assist with easy access to the original article.

The restricted use of colours and fonts was recognised by most of the participants, ranging between 79.3 and 86.2% (Table 1). Graphic design experts concurred that white, black, and basic colours are preferred, and that the background and font colours should be contrasted. Data and information that need to be emphasised must be highlighted with brighter colours. There should be a match if a combination of colours is used [16, 20].

With reference to Fig. 5 regarding the sources where participants find information presented as infographics, more than 65% of the participants make use of journal publications and the Internet. Only two mentioned “other” but did not provide the source. We published our infographics on figshare, an open-access repository where research outputs are available in a citable, shareable, and discoverable manner. We recognised the need to create a format that could be ready to use, especially adapted for the population of ophthalmologists involved with SBE. Online nano- and microlearning, social constructivism, workplace-based learning, competency medical education, and “just in time” learning/teaching can be seen as connected nodes underpinning infographics to maximise and augment faculty development opportunities [14, 30]. Microlearning as an educational strategy has demonstrated a positive effect on the knowledge and confidence of health professions students in performing procedures, retaining knowledge, studying, and engaging in collaborative learning [31]. There are several examples of microlearning using online education for faculty development under an online micromodule [32, 33], as an online application of infographics [34], and as online simulation-based sessions [35].

**Fig. 6:**
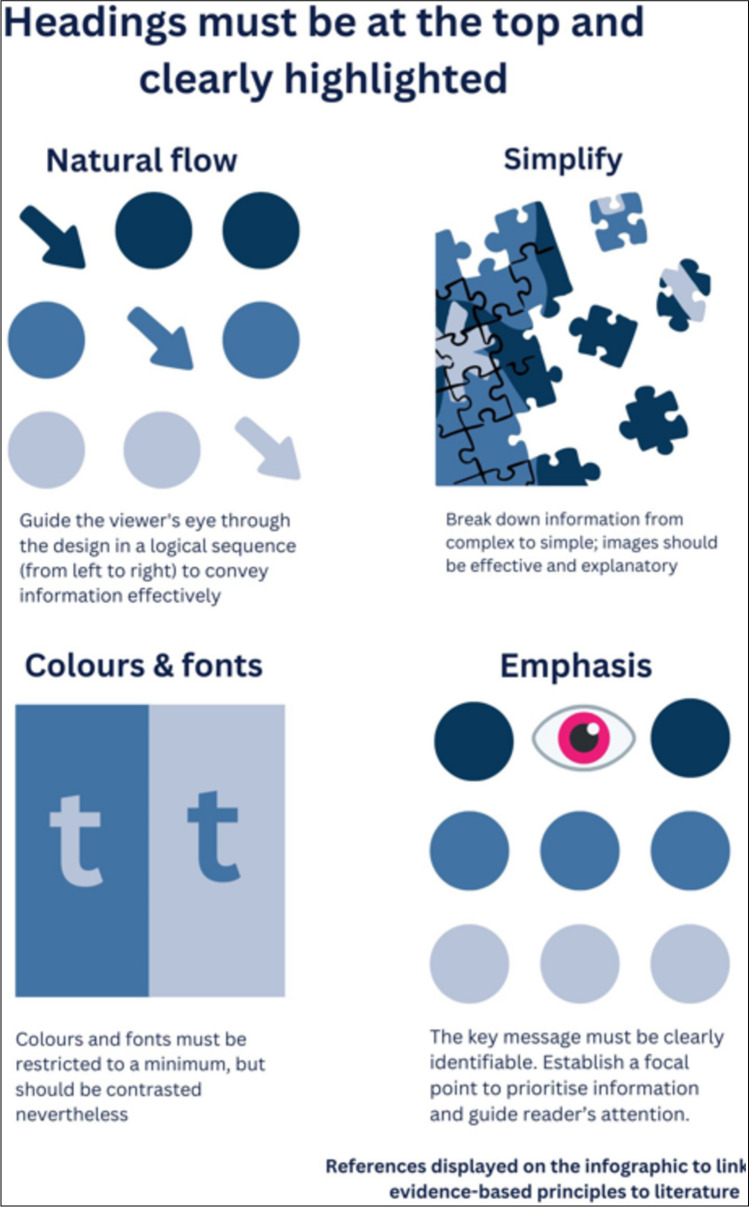
Recommendations for the development of infographics

Our findings lead us to suggest recommendations for an effective infographic that includes the following features summarised in Fig. 6. We recommend that the target audience must be clearly identifiable through words and images. A highlighted heading, preferably at the top of the infographic, is more effective. Follow a clear narrative through lines and arrows allowing natural flow from left to right. The key message or objective must be clearly identifiable. Transferability of information to teaching practice renders complex educational principles useful to facilitators. Evidence-based principles dictate clear links with literature via references displayed on the infographic and will assist users of the infographic to access the original article easily. To be more effective, colours and fonts must be restricted to a minimum. Elements must be aligned in a logical way prioritising key parts. Simple but effective imagery is recommended and if charts and graphs are used, it must be applicable and self-explanatory.

Dissemination of the infographics to the target audience can be through journal publications, social media, or Google Scholar. Many reputable journals such as *JAMA*, *NJEM*, and *BMJ* recommend that users cite the infographic, and it is advised that the infographic must have a DOI to promote its likelihood of being cited, shared, and discovered, or it can be used as tools for Just in Time Teaching (JiTT) or microlearning strategies. Infographics serve as valuable tools for enhancing teaching practices. These recommendations can assist educators in designing infographics, particularly when graphic designers are not available to provide support.

## Conclusion

We contend that outlining the value of SBE is relevant to building effective and enjoyable educational programs following best educational practices. Infographics, as pragmatic focused graphic storytelling tools, ready and quick to use and applied in the practice setting, can be very useful in the ophthalmologist educators’ daily teaching activities, and can also be integrated into SBE programs. Given their inherent qualities, infographics are particularly well-suited for nano- and microlearning strategies, serving as effective Just in Time Teaching (JiTT) tools to support professional development and competency-based education.

## Data Availability

Data are not available due to South African legislation on the protection of personal information.
